# Efficient Optical Sensing Based on Phase Shift of Waves Supported by a One-Dimensional Photonic Crystal

**DOI:** 10.3390/s21196535

**Published:** 2021-09-30

**Authors:** Roman Kaňok, Petr Hlubina, Lucie Gembalová, Dalibor Ciprian

**Affiliations:** Department of Physics, Technical University Ostrava, 17. Listopadu 2172/15, 708 00 Ostrava-Poruba, Czech Republic; lucie.gembalova@vsb.cz (L.G.); dalibor.ciprian@vsb.cz (D.C.)

**Keywords:** photonic crystal, interferometry, spectral domain, spatial domain, Bloch surface waves, guided waves, Kretschmann configuration, relative humidity of air

## Abstract

Interferometric methods of optical sensing based on the phase shift of the Bloch surface waves (BSWs) and guided waves (GWs) supported by a one-dimensional photonic crystal are presented. The photonic crystal, composed of six SiO_2_/TiO_2_ bilayers with a termination layer of TiO_2_, is employed in the Kretschmann configuration. Under resonance condition, an abrupt phase change is revealed, and the corresponding phase shift is measured by interferometric techniques applied in both the spectral and spatial domains. The spectral interferometric technique employing a birefringent quartz crystal is used to obtain interference of projections of *p*- and *s*-polarized light waves reflected from the photonic crystal. The phase shifts are retrieved by processing the spectral interferograms recorded for various values of relative humidity (RH) of air, giving the sensitivity to the RH as high as 0.029 rad/%RH and 0.012 rad/%RH for the BSW and GW, respectively. The spatial interferometric technique employs a Wollaston prism and an analyzer to generate an interference pattern, which is processed to retrieve the phase difference, and results are in good agreement with those obtained by sensing the phase shift in the spectral domain. In addition, from the derivative of the spectral phase shifts, the peak positions are obtained, and their changes with the RH give the sensitivities of 0.094 nm/%RH and 0.061 nm/%RH for the BSW and GW, respectively. These experimental results demonstrate an efficient optical sensing with a lot of applications in various research areas.

## 1. Introduction

Dielectric structures composed of alternating stratified media, referred to as the one-dimensional photonic crystals (1DPhCs) or the Bragg reflectors, are interesting for their optical properties. Due to a periodic modulation of the refractive index (RI), regions of abandoned light frequencies—the photonic band gaps—exist and within them, light is not allowed to propagate through the structures [1,2]. Thus, the 1DPhCs have a high reflectivity and are widely used as reflective coatings and filters [3]. The 1DPhCs can also act as planar waveguides [4] and the guided waves (GWs) can be used in sensing applications [5,6,7]. Last but not the least, the Bloch surface waves (BSWs) propagating along the interface of the 1DPhC with an external medium are widely used in sensing, including the angular [8,9,10,11] or wavelength [12,13,14,15,16,17] interrogations. Thus, the BSW-based sensors extend mature technologies applied in sensing that have several applications in different fields of biology [18], physics [18], and chemistry [18,19,20,21]. The BSW states exist within the band gap of a truncated 1DPhC. Both the GWs and BSWs cause a phase jump of incident light, similarly to the surface plasmon resonance (SPR). Therefore, sensors based on the phase detection of waves supported by a 1DPhC are feasible [22,23,24,25,26,27]. In addition, they represent an alternative to relative humidity sensors based on resonances of surface plasmons [16], BSWs [16], whispering gallery modes [28], guided modes [4,7,29], photonic crystal modes [30], and lossy modes [31,32].

One of the advantages of the BSWs supported by 1DPhCs is the fact that also *s*-polarized light can be used for their excitation, depending on the structure geometry. Since the structure is composed of dielectric materials, absorption is very low and resonance dips in the reflectance spectrum are narrow. In addition, because of its chemical stability, sensing using the 1DPhCs can be adopted in aggressive environments. Although the sensing using the reflectance evaluation in the spectral domain is more often reported owing to a simple set-up, the phase interrogation has some substantial advantages even if the method is more complex. One of them is that resonance phase peaks are narrower than the resonance reflection dips. Moreover, in many cases the resonance reflection dips cannot be resolved and an interferometric method to measure an abrupt phase change overcomes the limitation.

In this paper, two optical interferometric sensing methods based on measurement of the phase shift of the BSWs and GWs supported by a 1DPhC are presented. The methods, as alternatives to original approaches [33,34,35,36,37,38,39], are applied in both the spectral and spatial domains, and as an analyte, moist air of a varied relative humidity (RH) is used. For the 1DPhC under test we show that the phase shifts of both the BSW and GW can be resolved using the spectral method. On the contrary, only the phase shift of the GW can be resolved using the spatial method. At a specific wavelength, the phase shift is determined as a function of the RH. The sensor performance is evaluated in terms of sensitivity, and in the case of the BSWs, achieved sensitivity to the humidity is as high as 0.029 rad/%RH. Similarly, in the case of the GWs, the sensitivity reaches 0.012 rad/%RH. Moreover, to show an advantage of the spectral method, derivative of the phase shift is performed, and peak position is tracked as a function of the RH, giving the sensitivity to the RH as high as 0.094 nm/%RH and 0.061 nm/%RH for the BSW and GW, respectively.

The paper is organized as follows. The first part is focused on the material characterization. In the second part, computational tools used in theoretical model are introduced. The third part is focused on a band structure of an infinite 1DPhC. Then, the theoretical results are presented. In the following part, an experimental set-up used in recording the interferograms is described. The last but one part is focused on experimental results obtained by techniques applied in both the spectral and spatial domains. In the final part, conclusions are presented.

## 2. Theoretical Model

### 2.1. Material Characterization

**Figure 1:**
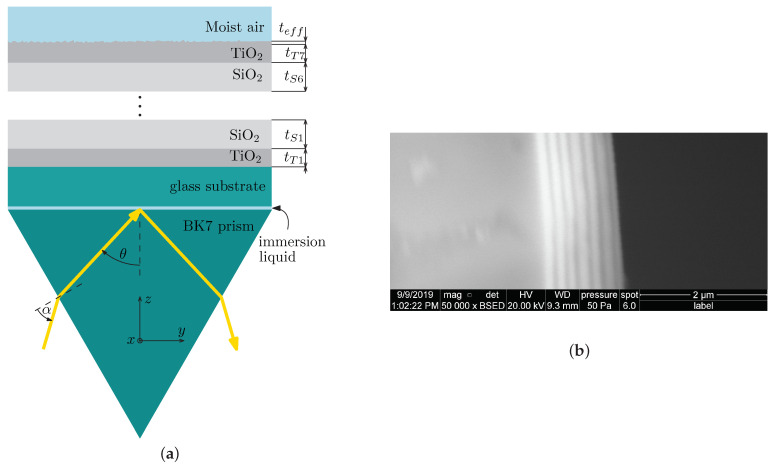
(**a**) A coupling prism with a photonic crystal under consideration. (**b**) SEM image of the photonic crystal.

The multilayer structure under study is shown in Figure 1a, and it represents a 1DPhC consisting of six SiO_2_/TiO_2_ bilayers and a termination layer of TiO_2_. The 1DPhC is deposited on a glass substrate and employing an immersion oil, the substrate is attached to a coupling prism made of BK7 glass in the Kretschmann configuration. In Figure 1b, a detail image of the structure profile obtained by a scanning electron microscope (SEM) is shown, revealing different layer thicknesses. To characterize the thin layers, the variable angle spectroscopic ellipsometry (VASE) measurement was employed. Data obtained by the VASE were processed using the CompleteEASE software (J.A. Woollam Co., Inc., Shanghai, China) and the thicknesses of the layers were determined, as summarized in Table 1.

Moreover, the RI dispersions of the layers and of the substrate were also determined as a result of fitting the data obtained by the VASE. In the case of the glass substrate, the RI as a function of wavelength is expressed by Cauchy formula

(1) $$n_{sub}\left(\lambda \right)=A-B\lambda +C\lambda^{2}-D\lambda^{3},$$

where values of constants obtained by the VASE are A=1.51824, *B* = 0.19112 μm^−1^, C=0.019391 $\mu m^{-2}$ and D=0.07108 $\mu m^{-3}$, when wavelength λ is in micrometers. The RI dispersion of thin films is described by formula

(2) $$n_{i}^{2}\left(\lambda \right)=A+\frac{B\lambda^{2}}{\lambda^{2}-C^{2}}-D\lambda^{2},$$

where A,B,C and *D* are constants and *i* = TiO_2_, SiO_2_ indicates the material. Their values obtained by the VASE for TiO_2_ are *A* = 0, *B* = 4.672, *C* = 0.22935 μm, *D* = 0 $\mu m^{-2}$. The constant values obtained for SiO_2_ are *A* = 1.348, *B* = 0.756, *C* = 0.10683 μm, *D* = 0.00975 μm^−2^. The obtained relations are valid in a wavelength range from 376 nm to 1700 nm. Similarly, the RI of the BK7 prism is described by a three term Sellmeier formula specified elsewhere [40], valid in a wavelength range from 0.3 μm to 2.5 μm.

**Figure 2:**
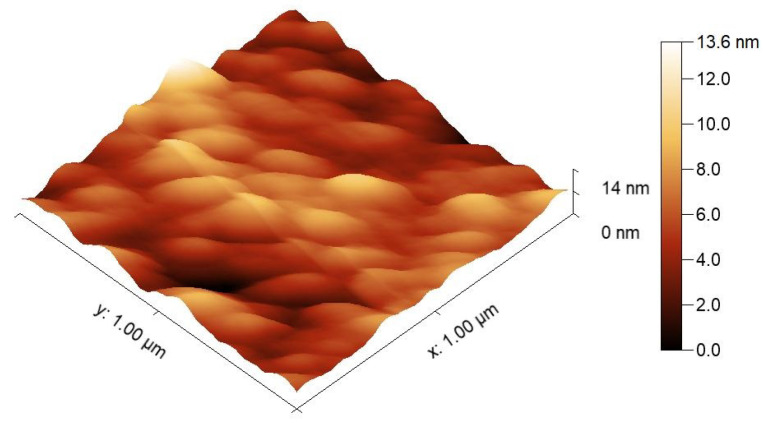
A topography image obtained by an AFM measurement.

At the top of the termination layer, there is a rough surface. To confirm the rough surface, a square of 10 μm × 10 μm chosen on the surface was inspected by the atomic force microscopy (AFM) and data obtained were processed using Gwyddion software. A topography image of the 1DPhC surface corrected by a flat surface subtraction is shown in Figure 2, and the average roughness $R_{a}=\left(1.302\pm 0.37\right)$ nm was obtained.

Under assumption that the average roughness is smaller than the wavelength of interacting light, the rough surface can be approximated by a layer of an effective medium. In the case of the Bruggeman effective media approximation (EMA), the dielectric constant $\epsilon_{eff}$ describing the layer satisfies the equation

(3) $$f\frac{\epsilon_{a}-\epsilon_{eff}}{\epsilon_{a}+2\epsilon_{eff}}+\left(1-f\right)\frac{\epsilon_{b}-\epsilon_{eff}}{\epsilon_{b}+2\epsilon_{eff}}=0,$$

where $\epsilon_{a}$ end $\epsilon_{b}$ are dielectric constants of media *a* and *b*, respectively, and f∈<0,1> is a fraction of medium *a* in the effective medium layer. By default, set-up of the CompleteEASE software, 50% of void is assumed (*f* = 0.5, $\epsilon_{b}$ = 1) in the models.

The multilayer detection structure is sensitive to changes in RI of the external medium (analyte) of the 1DPhC. Since the change in the RI of moist air due to RH change is very low ($\Delta \mathrm{RI}\approx 3.6\times 10^{-7}$ obtained [41] for wavelength λ = 532 nm, temperature *t* = 20 C, atmospheric pressure *p* = 1013.25 hPa and RH change from 30% to 80%), the mechanism of sensitivity of the proposed sensor to moist air (see next Sections) has to be caused by other phenomena. One of them is adsorption of water molecules on the rough surface [42,43] of the 1DPhC. To gain insight into the effect, we simulate it by involving contribution of dielectric function of water in calculations of the Bruggeman EMA. It can be done using of Equation (3) recursively. First, the RI dispersion of water can be described by a four term Sellmeier formula [44]

(4) $$n_{w}^{2}\left(\lambda \right)\;=\;1+\sum_{n=1}^{4}\frac{A_{i}\lambda^{2}}{\lambda^{2}-\lambda_{i}^{2}},$$

where the constants $A_{i}$ and $\lambda_{i}^{2}$ valid for temperature of 20 °C are $A_{1}=5.684027565\times 10^{-1}$, $A_{2}=1.726177391\times 10^{-1}$, $A_{3}=2.086189578\times 10^{-2}$, $A_{4}=1.130748688\times 10^{-1}$, $\lambda_{1}^{2}=5.101829712\times 10^{-3}$ μm^2^, $\lambda_{2}^{2}=1.821153936\times 10^{-2}$ μm^2^, $\lambda_{3}^{2}=2.620722293\times 10^{-2}$ μm^2^, $\lambda_{4}^{2}=1.069792721\times 10$ μm^2^.

Then the dielectric function $\epsilon_{eff}^{\prime }$ containing contributions of air and water is obtained using Equation (3), considering $\epsilon_{a}=n_{w}^{2}$ and $\epsilon_{b}=1$ for fraction of medium *a* (water) f∈<0,1>, where for $f=0\;\to \;\epsilon_{eff}^{\prime }=\epsilon_{a}$ and for $f=1\;\to \;\epsilon_{eff}^{\prime }=\epsilon_{w}$. After that, the final dielectric function $\epsilon_{eff}$ involving contributions of TiO_2_ and previously determined effective medium is obtained, considering $\epsilon_{a}=n_{TiO_{2}}^{2}$ and $\epsilon_{b}=\epsilon_{eff}^{\prime }$, with fixed f=0.5.

### 2.2. Matrix Formalism

**Figure 3:**
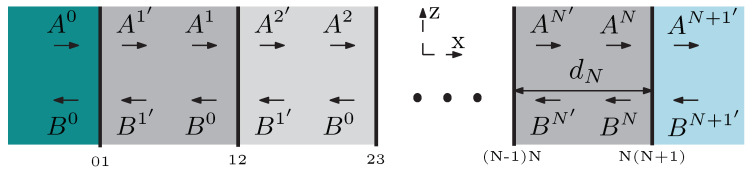
A structure of *N* layers under study.

Interaction of electromagnetic waves with dielectric periodic structures can be effectively described by the 2×2 matrix method [45,46], assuming that the media are homogeneous and isotropic. First, a structure of *N* layers sandwiched between two semi-finite media is considered, as shown in Figure 3. Amplitudes of right and left propagating plane waves are represented by $A^{i}$ and $B^{i}$, respectively, while the superscript ′ indicates that the wave is at the left boundary of the layer.

The corresponding right and left propagating modes can be represented as column vectors, and these vectors at the two sides of the interface ij are related via so-called dynamic matrix $D_{ij}$

(5) $$\left(\begin{array}{c} A^{i}\\ B^{i} \end{array}\right)=D_{ij}\left(\begin{array}{c} A^{j^{\prime }}\\ B^{j^{\prime }} \end{array}\right),$$

where

(6) $$D_{ij}=\frac{1}{t_{ij}}\left(\begin{array}{c} 1\;\;\;r_{ij}\\ r_{ij}\;\;\;1 \end{array}\right),$$

and $r_{ij}$ and $t_{ij}$ are the reflection and transmission coefficients of the ij-th interface, respectively. These coefficients are given by

(7) $$r_{ij}=\left\{\begin{array}{cc} \frac{k_{ix}-k_{jx}}{k_{ix}+k_{jx}} & \mathrm{for}\;s-\mathrm{pol}.\;\mathrm{wave},\\ \frac{n_{i}^{2}k_{jx}-n_{j}^{2}k_{ix}}{n_{i}^{2}k_{jx}+n_{j}^{2}k_{ix}} & \mathrm{for}\;p-\mathrm{pol}.\;\mathrm{wave}, \end{array}\right.$$

and

(8) $$t_{ij}=\left\{\begin{array}{cc} \frac{2k_{ix}}{k_{ix}+k_{jx}} & \mathrm{for}\;s-\mathrm{pol}.\;\mathrm{wave},\\ \frac{2n_{i}^{2}k_{jx}}{n_{i}^{2}k_{jx}+n_{j}^{2}k_{ix}} & \mathrm{for}\;p-\mathrm{pol}.\;\mathrm{wave}, \end{array}\right.$$

where $k_{ix}=k_{0}{\left[{\left(n_{l}\right)}^{2}-{\left(n_{0}\sin\theta \right)}^{2}\right]}^{1/2}$ is the normal component of the wave vector of the light wave in the *i*-th medium. When a wave propagates through the *i*-th layer, a phase change $k_{l}t_{l}$ or $-k_{l}t_{l}$ is introduced, depending on whether the wave is right or left propagating, respectively. Thus, the modes on the two side boundaries of the *i*-th layer are related via

(9) $$\left(\begin{array}{c} A^{j^{\prime }}\\ B^{j^{\prime }} \end{array}\right)=P_{i}\left(\begin{array}{c} A^{j}\\ B^{j} \end{array}\right),$$

where

(10) $$P_{l}=\left(\begin{array}{c} e^{ik_{l}t_{l}}\;\;\;\;0\\ 0\;\;\;\;e^{-ik_{l}t_{l}} \end{array}\right)$$

is the propagation matrix. Putting all this together, a matrix equation that relates the wave amplitudes in substrate and superstrate can be obtained

(11) $$\left(\begin{array}{c} A^{0}\\ B^{0} \end{array}\right)=M\left(\begin{array}{c} A^{N+1}\\ B^{N+1} \end{array}\right),$$

where the overall transfer matrix is expressed as

(12) $$M=\left(\begin{array}{c} M_{11}M_{12}\\ M_{21}M_{22} \end{array}\right)=\left[\prod_{i=1}^{N}D_{(i-1)i}P_{i}\right]D_{N(N+1)}.$$

The complex reflection coefficient of the structure is a ratio of the reflected wave amplitude $B^{0}$ to the incident wave amplitude $A^{0}$. Assuming that no light is incident from the superstrate ($B^{N+1^{\prime }}=0$), using Equation (12) we obtain

(13) $$r_{s,p}=\left|r_{s,p}\right|e^{i\delta_{p,s}}=\frac{M_{21}}{M_{11}},$$

The phase difference between *p*- and *s*-polarized light waves can thus be determined as $\Delta \left(\lambda \right)=\delta_{p}\left(\lambda \right)-\delta_{s}\left(\lambda \right)$.

Reflectance of the structure can be expressed as a squared modulus of the coefficient

(14) $$R_{s,p}={\left|r_{s,p}\right|}^{2}.$$

### 2.3. Band Structure

**Figure 4:**
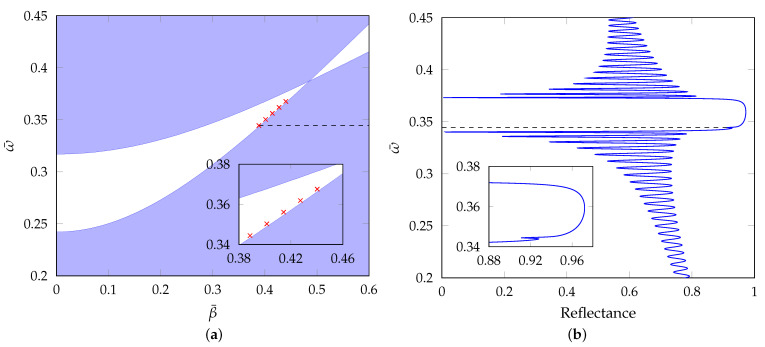
(**a**) A band diagram of an infinite multilayer structure. Red crosses are related to the Bloch states. Inset shows positions of the resonance states in detail. (**b**) Reflectance of the structure with 100 bilayers as a function of $\bar{\omega }$. Light is *p*-polarized, angle of incidence is θ=48°. Inset shows a shallow resonance dip related to the Bloch surface wave.

To understand the shape of the reflection spectra, the band structure concept, similar to the solid-state physics, can be used. Considering an infinite 1DPhC, the periodicity leads to existence of allowed and forbidden bands—the waves at some frequencies can propagate through the 1DPhC, whereas some other cannot. Using the 2×2 matrix formalism described in the previous section, the transmission matrix that links electric field amplitudes at the input and at the output of one bilayer (one unit cell) of a periodic structure can be obtained. Under assumption that the RI in the 1DPhC is periodically modulated, the Bloch’s theorem can be applied, which states that a solution of the wave equation has a form of a plane wave modulated by a periodic function with the same period as the RI. In resulting eigenproblem, eigenvalues of the transmission matrix are related to the Bloch wave number *K*. For derivation, see [45] and resulting equation for *p*-polarized light is [16]

(15) $$\cos\left(K\Lambda \right)=\cos\left(k_{a⊥}a\right)\cos\left(k_{b⊥}b\right)-\frac{1}{2}\left(\frac{n_{b}^{2}k_{a⊥}}{n_{a}^{2}k_{b⊥}}+\frac{n_{a}^{2}k_{b⊥}}{n_{b}^{2}k_{a⊥}}\right)\sin\left(k_{a⊥}a\right)\sin\left(k_{b⊥}b\right),$$

where *a*, *b* and Λ are thicknesses of the layers and a bilayer thickness, respectively, $k_{i⊥}=\sqrt{{\left(\frac{n_{i}\omega }{c}\right)}^{2}-\beta^{2}}$, i=a,b is a normal component of a wave vector in corresponding medium and β is a propagation constant. Equation (15) gives dependence of the propagation constant β on the angular frequency ω. Regions where cos(KΛ)<1 are related to propagating waves (real *K*). In the case of cos(KΛ)>1, the waves are evanescent (imaginary *K*). In Figure 4a, a band diagram of the infinite structure composed of SiO_2_/TiO_2_ bilayers is shown. Here, reduced variables $\bar{\beta }=\beta \frac{\Lambda }{2\pi }$ and $\bar{\omega }=\frac{\omega }{c}\frac{\Lambda }{2\pi }$ were used and as thicknesses of the SiO_2_ and TiO_2_ layers, arithmetic means of the thickness values obtained by the VASE were used (*a* = 115 nm, *b* = 78 nm). The white region represents a photonic band gap, while the blue regions represent the allowed bands. The red crosses are related to surface waves excited on a finite 1DPhC consisting of 100 SiO_2_/TiO_2_ bilayers (when substrate is glass and superstrate is air) for various angles of incidence. It can be seen that their positions are in the photonic band gap and thus they are identified as the Bloch surface waves.

In Figure 4b, the reflectance of the structure with 100 bilayers of SiO_2_/TiO_2_ as a function of $\bar{\omega }$ is shown. The 2×2 matrix formalism was used in calculating the reflectance, assuming approximate extinction coefficients for TiO_2_ and SiO_2_ layers of $k_{TiO_{2}}=1.6\times 10^{-3}$ and $k_{SiO_{2}}=3.4\times 10^{-4}$, respectively [16,17]. This figure clearly shows that the Bloch surface wave resonance shows up as a shallow dip in the reflectance spectrum.

## 3. Theoretical Results

**Figure 5:**
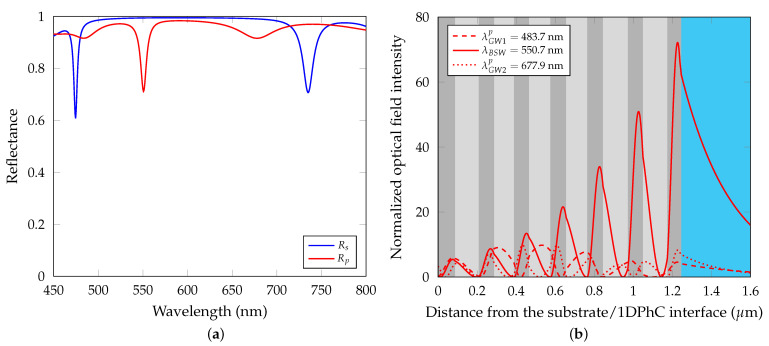
(**a**) Theoretical reflectance of the 1DPhC as a function of wavelength for both *p* and *s*-polarized light. The dip at a wavelength of approximately 551.5 nm is associated with the Bloch surface wave. (**b**) Normalized optical field distribution of *p*-polarized light in the structure. Angle of incidence θ=41.9°.

To gain quantitative understanding of the reflection spectra, the reflectance of the 1DPhC was computed in the wavelength domain, as shown in Figure 5a, using the 2×2 matrix method. The extinction coefficients $k_{TiO_{2}}$ and $k_{SiO_{2}}$ given in the previous section were considered in the calculations, to enlarge resonance dips occurring in the reflectance spectra (otherwise they would be not observable). In the case of *p*-polarized light, three resonance dips are observed in the given wavelength region, a narrow dip approximately at $\lambda_{BSW}=551.5$ nm corresponds to the BSW, while broad dips at $\lambda_{GW1}^{p}=483.7$ nm and $\lambda_{GW2}^{p}=677.9$ nm, respectively, correspond to guided waves. This is supported by the normalized optical field distribution of *p*-polarized light in the 1DPhC shown in Figure 5b.

The optical field is proportional to $|H_{y}{|}^{2}$, where $H_{y}$ is magnetic field component. The enhanced optical field intensity at the wavelength $\lambda_{BSW}$ corresponds to character of the BSW field with exponential envelopes, while the intensities at wavelengths $\lambda_{GW1}^{p}$ and $\lambda_{GW2}^{p}$ are enhanced inside the structure and thus their characters correspond to guided waves. In the case of *s*-polarized light, two resonance dips are observed in the Figure 5a at wavelengths $\lambda_{GW1}^{s}=474.5$ nm and $\lambda_{GW2}^{s}=735.1$ nm, delimiting the borders of the photonic band gap of *s*-polarized light.

**Figure 6:**
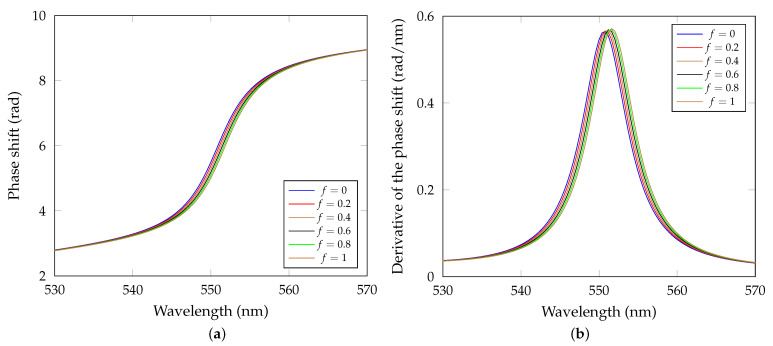
(**a**) Theoretical phase shift as a function of the wavelength with the increasing fraction of water *f* in the effective medium layer. (**b**) Derivative of the phase shift as a function of wavelength.

The theoretical response of the proposed sensor can be shown for different water amount adsorption on the rough surface of the 1DPhC, although the dependence on the RH change is unknown. In Figure 6a, the phase response of the sensor to filling the rough surface with water is shown for water fractions *f* = 0, 0.2, 0.4, 0.6, 0.8 and 1. As can be seen, a red shift occurs for higher water fraction *f*. Derivative of the phase shift as a function of wavelength is shown in Figure 6b. Extreme point of the derivative is related to the so-called resonance wavelength $\lambda_{R}$ at which the BSW is excited.

## 4. Experimental Set-Up

**Figure 7:**
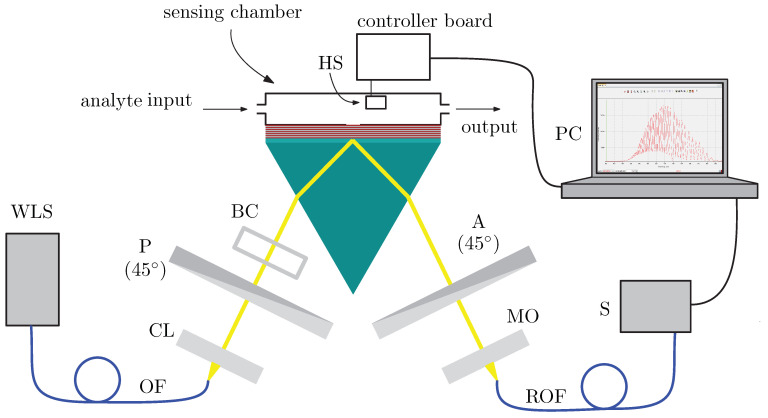
Experimental set-up consisting of white-light source WLS, optical fiber OF, collimating lens CL, polarizer P, birefringent crystal BC, a coupling prism with a multilayer structure and a sensing chamber, humidity sensor HS, analyzer A, microscope objective MO, read optical fiber ROF, spectrometer A and personal computer PC.

To measure phase shifts of waves under resonance conditions, interferometric techniques applied in both the spectral and spatial domains are employed. In Figure 7, an experimental set-up employing the spectral interferometric technique is shown. A composition of the set-up and a measurement procedure are described in the following part. A light beam is generated by white-light source WLS (halogen lamp HL-2000, Ocean Optics, Dunedin, FL, USA), guided by optical fiber OF and then it passes through collimating lens CL. The collimated beam (of diameter approximately 1 mm) passes through linear polarizer P (LPVIS050, Thorlabs, Newton, MA, USA), with optical axis-oriented 45° with respect to the plane of incidence, and both polarization components *s* and *p* are generated. An optical path difference between the components is introduced by birefringent quartz crystal BC of thickness *d* = 6 mm, so that interference fringes have appropriate period in resulting interferograms. The light beam then reflects from the multilayer structure which was prepared by a method of sputtering (Meopta, Přerov, Czech Republic), primarily made as a Bragg reflector.

**Figure 8:**
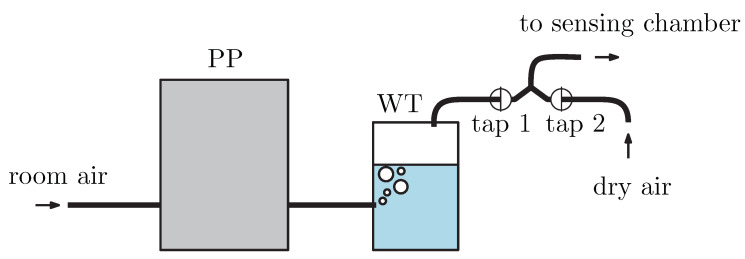
System to control the RH of air, including peristaltic pump PP and water tank WT.

A linear polarizer used as analyzer A (LPVIS050, Thorlabs) with optical axis-oriented 45° with respect to the plane of incidence projects the polarization components into one direction of polarization, so they may interfere. Then, the light beam is launched by microscope objective MO into read optical fiber ROF (M15L02, Thorlabs) and then led to the spectrometer (USB4000, Ocean Optics). As a result of the procedure, a spectral interferogram is obtained. The angle of incidence on the air/prism interface is adjusted to be α=24° (see Figure 1). The analyte is moist air with different RHs approximately from 35% to 80%. To control the RH, a system including peristaltic pump PP (BT100M, 2xYZ1515x, Baoding Chuang Rui Precision Pump Co., Ltd., Baoding, China) and water tank WT is employed (see Figure 8). To increase the RH, room air is pushed by the PP to the WT (bellow the water level) and humidified air flows from the WT to the sensing chamber, when tap 1 is opened and tap 2 is closed. To decrease the RH, room air is injected by a fan and flows to the sensing chamber, while tap 2 is opened and tap 1 is closed. The air is dried using a moisture absorber at the entrance of the line. The lowest RH is thus given by the RH of the room air. To check the RH value, an electric, commercially available sensor based on Arduino system (Arduino UNO, Ivrea, Italy) is employed in the chamber and is connected with the computer.

The second method, the spatial-domain interferometric method, was described in detail in a previous paper [47] demonstrating measurement of changes in the RI of a liquid analyte. In that method we used the experimental set-up comprising a laser diode irradiating at wavelength $\lambda_{LD}=$ 637.1 nm, launching optics, a gold coated SF10 glass plate (Accurion, Goettingen, Germany), a coupling prism (SF10 glass, Accurion, Goettingen, Germany), a Wollaston prism (WP05, Thorlabs, USA) and a CCD camera (PL-B952U, Pixelink, Ottawa, Canada). For solutions of distilled water with ethanol in various weight ratios, spatial fringe patterns were recorded and phases as a function of pixel number were retrieved from intensity for a single row of the patterns. After replacing the plasmonic structure with the 1DPhC on the glass substrate, the interferometric method applied in the spatial domain can also be used for measuring the phase shift for the BSWs and GWs.

## 5. Experimental Results

First, we employed a spectral interferometric technique and measured the spectra of light reflected from the 1DPhC under test in both *s* and *p* polarizations, and resolved no dips. This is due to the extinction coefficients $k_{TiO_{2}}$ and $k_{SiO_{2}}$ that are smaller than those used in the theoretical simulations. Based on the theoretical results, we proceeded to obtain the phase responses.

**Figure 9:**
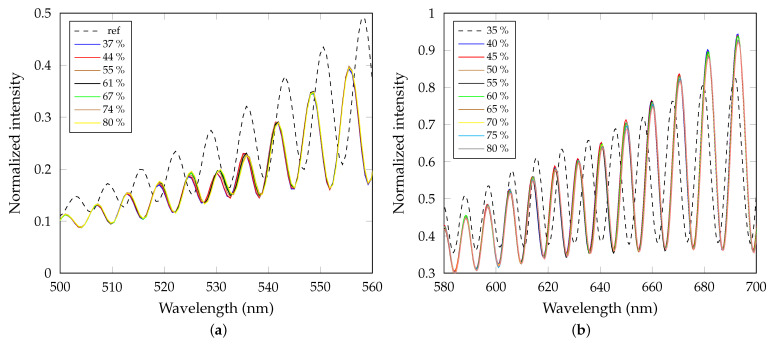
(**a**) Measured spectral interferograms involving the phase change of the BSW together with the reference one. (**b**) Measured spectral interferograms involving the phase change of the GW2 together with the reference one.

Following the measurement procedure described in the previous section, the phase shift is retrieved from two spectral interferograms using a procedure presented in a previous paper [48]. A reference and the BSW-based interferograms were recorded, as shown in Figure 9a. The reference interferogram was obtained in the set-up without the multilayer structure (BSW resonance does not occur). A shift of the fringes can be observed around wavelength λ=532 nm. In Figure 9b, interferograms involving the phase change of the GW2 are shown.

### 5.1. The BSW-Based Response

**Figure 10:**
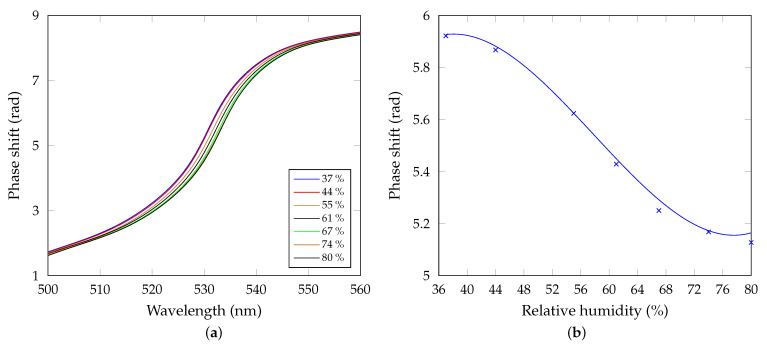
(**a**) Measured phase shift as a function of the wavelength for the increasing air humidity. (**b**) Measured phase shift as a function of the relative humidity with a polynomial fit (R^2^ = 0.9965). Wavelength is λ=532 nm.

Using the WFT, the phase functions $\Phi \left(\lambda \right)=\Delta_{BC}\left(\lambda \right)+\Delta \left(\lambda \right)$ and $\Phi_{R}\left(\lambda \right)=\Delta_{BC}\left(\lambda \right)+\Delta_{R}\left(\lambda \right)$ are obtained from the interferograms, where $\Delta \left(\lambda \right)=\delta_{s}\left(\lambda \right)-\delta_{p}\left(\lambda \right)$ is phase difference between *s* and *p*-polarized waves, when the BSW is excited, and $\Phi_{R}\left(\lambda \right)$ is the reference phase difference. Finally, the phase shift $\delta_{\mathrm{BSW}}\left(\lambda \right)=\Delta \left(\lambda \right)-\Delta_{R}\left(\lambda \right)$ can be determined. Results are shown in Figure 10a and comparing them with the theoretical results shown in Figure 6a, we confirm good correspondence. The abrupt phase change in the wavelength region approximately located from 520 nm to 545 nm is related to the BSWs and a red shift is observed for increasing RH. Phase shift as a function of the RH at a specific wavelength is shown in Figure 10b. The wavelength λ = 532 nm was chosen to obtain the highest changes of the phase shift with the RH. The polynomial fit (blue line) represents the sensor response curve, and the response is linear approximately in a range of 45–70 %RH.

**Figure 11:**
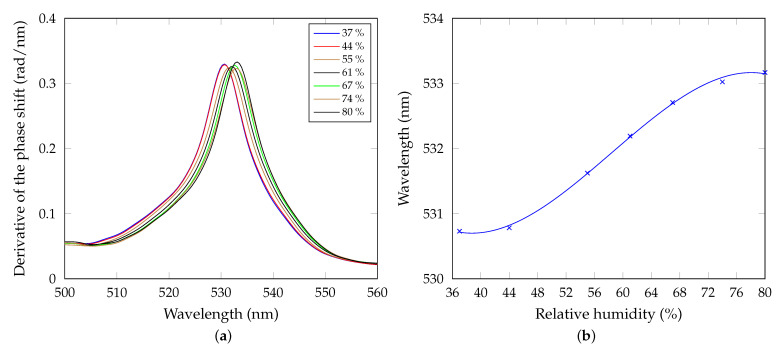
(**a**) Derivative of the phase shift as a function of the wavelength for the increasing air humidity. (**b**) Wavelength of the extreme of the derivative of the phase shift as a function of the relative humidity with a polynomial fit (R^2^ = 0.99765).

The sensor performance is evaluated in terms of sensitivity. The sensitivity is defined as change of the output quantity with respect to the input quantity [49]. In this case, such parameters are the phase shift $\delta_{BSW}$ and the RH, respectively, so that the sensitivity is

(16) $$S_{\delta }=\left|\frac{\partial \delta_{BSW}}{\partial \mathrm{RH}}\right|$$

as a simple derivative of the response curve with respect to the RH. In our case, the sensitivity can be approximated using a quadratic function of the RH with its maximum value of 0.029 rad/%RH for the RH of approximately 58%. In addition, the derivative of the phase shift as a function of the wavelength for different air humidities is shown in Figure 11a.

Positions of the derivative maxima correspond to the resonance wavelengths $\lambda_{r}$ on which the BSWs are excited. These wavelengths can also be expressed as a function of the RH, as shown in Figure 11b, and a red shift in accordance with the theory is confirmed. Additionally here, the sensor response curve can be represented by a polynomial fit, which can be linearized approximately in a range of 45–70 %RH. In this case, the sensitivity to humidity can be expressed as derivative of the sensor response curve, represented by the resonance wavelength $\lambda_{r}$ versus the RH, with respect to the RH

(17) $$S_{\lambda }=\left|\frac{\partial \lambda_{r}}{\partial \mathrm{RH}}\right|.$$

The sensitivity can be also approximated by a quadratic function of RH. Its maximum value is $S_{\lambda }=0.094$ nm/%RH for the RH of approximately 58.40%. The sensor with the sensitivity value outperforms or is comparable with some of optical RH sensors employing different materials and methods, as presented in Table 2. In addition, the sensitivity depends on the structure geometry and surface porosity [30] and can be easily enhanced.

Although the sensitivity gives a comparable information about a sensor response, it does not involve influence of dip or peak width on the extreme point resolving in sensing methods using wavelength interrogation. For this reason, figure of merit defined as

(18) $$\mathrm{FOM}=\frac{S_{\lambda }}{\mathrm{FWHM}}$$

is used, where FWHM is full width at half maximum of the peak. For the BSW with FWHM of 10.93 nm, the FOM is as high as $8.6\times 10^{-3}$ %RH^−1^.

### 5.2. The GW-Based Response

**Figure 12:**
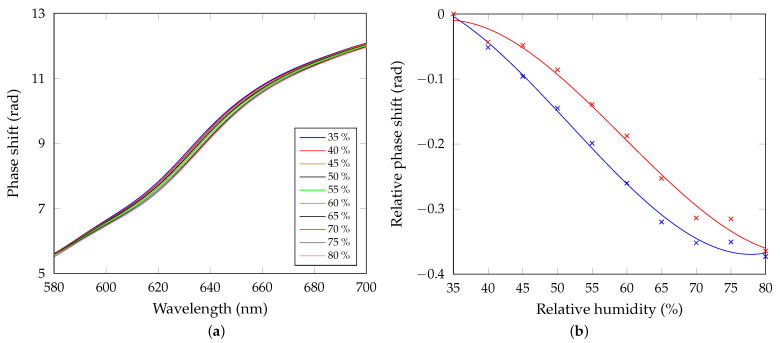
(**a**) Measured phase shift of the GW2 as a function of the wavelength for the increasing air humidity. (**b**) Measured relative phase shift as a function of the RH obtained by the spectral (blue) and spatial (red) phase detection methods with polynomial fits, $R^{2}=0.9964$ (blue), $R^{2}=0.9909$ (red).

Additionally, the GWs of the 1DPhC can be used in sensing applications. In Figure 12a, the phase shift of GW2 as a function of wavelength for different RHs is shown. As with the BSWs, an abrupt phase shift change can be observed, and a red shift occurs for the increasing RH. The phase shift relative to the reference (RH of 30%) as a function of the RH is shown in Figure 12b. The sensitivity is obtained using equation analogous to Equation (16), and once again a quadratic function was revealed, and the sensitivity reaches 0.012 rad/%RH for the RH of approximately 51.8%. As with the previous case, spectral derivatives were determined, and wavelengths of their extreme points depend on the RH of air. The sensitivity defined by Equation (17) was determined and it reaches 0.061 nm/%RH. The FWHM is now of approximately 38.7 nm and the FOM, defined by Equation (18), is as high as $1.6\times 10^{-3}$ %RH^−1^. Comparing the sensing using BSW and GW, the BSW-based sensing has higher sensitivity and FOM.

**Figure 13:**
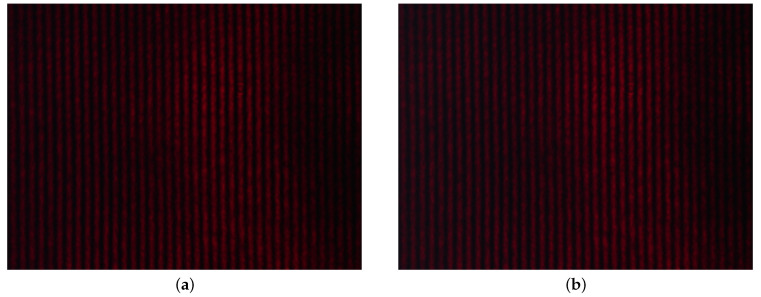
Recorded interference pattern obtained for the RH of 35% (**a**) and 80% (**b**).

Next, we employed a spatial interferometric technique to record the interference pattern under the condition that an appropriate angle of incidence of the light beam on the 1DPhC is adjusted, and the excitation wavelength of the second guided wave $\lambda_{GW2}^{p}$ matches the wavelength of the laser diode $\lambda_{LD}$. The results obtained for the RHs of 35% and 80%, respectively, are shown in Figure 13a,b. The relative phase shift from the first measured value (for the lowest RH) as a function of the RH is shown in Figure 12b, together with the results obtained from the spectral interferometric method. Good correspondence is achieved comparing the results. In this case, the sensitivity, defined by Equation (16), has a maximum value of 0.011 rad/%RH for the RH of approximately 59.5%.

Comparing the results from both methods, good correspondence was achieved. The spatial method is more demonstrative, but it has some limitations. These include availability of an LD source irradiating at the resonance wavelength, and difficulties in obtaining the spatial fringes of required separation, as the angle between the interfering beams needs to be adjusted. In the case of the spectral method, the fringes period can be varied by changing the thickness of the BC only. Additionally, it is an advantage of the spectral method that the wavelength interrogation can be used, yielding a high FOM.

## 6. Conclusions

In this paper, phase shift detection methods based on interferometry applied in both the spectral and spatial domains have been presented. The methods have been employed for a 1DPhC composed of six SiO_2_/TiO_2_ bilayers with a termination layer of TiO_2_, and the phase shifts of both the BSWs and GWs were measured. First, theoretical analysis has been performed for the 1DPhC attached to a coupling prism in the Kretschmann configuration. It was revealed that the waves excited under resonance conditions are accompanied by an abrupt phase change. Next, the 1DPhC has been analyzed experimentally in the spectral domain in measuring the phase shift induced by both the BSW and GW for the RH of moist air in a range of 37–80 %RH. The phase shift changes linearly with the RH in a region of approximately 45–70 %RH, and the highest achieved sensitivity is 0.029 rad/%RH.

In addition, the derivative of the spectral phase shifts have been evaluated to obtain the peak positions, and their changes with the RH gave the sensitivities of 0.094 nm/%RH and 0.061 nm/%RH for the BSW and GW, respectively. The 1DPhC has been analyzed experimentally in the spatial domain as well, but the phase shift was able to measure for the GW only. The corresponding sensitivity to the RH of 0.011 rad/%RH agrees well with that obtained by the spectral method. These experimental results demonstrate an efficient optical sensing employing the 1DPhC. The use of the interferometric methods with the phase shift retrieval can be extended to other 1DPhCs to manifest a lot of applications in various research area. As an example, detailed theoretical and experimental analyses of adsorption properties of 1DPhCs can be performed. In addition, the results are important from point of view of new sensors development and design.

## Figures and Tables

**Table 1:** Thin layer thicknesses obtained by the VASE.

Layer	Thickness (nm)	Layer	Thickness (nm)
$t_{T1}$	87.65	$t_{S1}$	120.21
$t_{T2}$	79.09	$t_{S2}$	101.75
$t_{T3}$	77.28	$t_{S3}$	109.24
$t_{T4}$	80.74	$t_{S4}$	108
$t_{T5}$	80.89	$t_{S5}$	127.3
$t_{T6}$	76.85	$t_{S6}$	125.02
$t_{T7}$	64.41	$t_{eff}$	6.96

**Table 2:** Comparison of different optical RH sensors.

Material	Method	RH Range	Sensitivity (nm/%RH)	Ref.
plasmonic multilayer	surface plasmon wave resonance	20–80%	0.072	[16]
dielectric multilayer	surface Bloch wave resonance	22–80%	0.065	[16]
polymer coating	whispering gallery mode resonance	0–60%	0.013	[28]
agarose gel	guided mode resonance	20–80%	0.150	[4]
porous thin film	photonic crystal mode resonance	11–84%	0.296	[30]
indium tin oxide	lossy mode resonance	65–90%	0.212	[31]
copper oxide	lossy mode resonance	30–90%	0.636	[32]
